# The Effect of Vacuum Deep Frying Technology and *Raphanus sativus* on the Quality of Surimi Cubes

**DOI:** 10.3390/foods10112544

**Published:** 2021-10-22

**Authors:** Jinghao Chen, Yi Lei, Jiaxin Zuo, Zebin Guo, Song Miao, Baodong Zheng, Xu Lu

**Affiliations:** 1 College of Food Science, Fujian Agriculture and Forestry University, 15 Shangxiadian Road, Fuzhou 350002, China; jinghaochen92@yeah.net (J.C.); leiyi1220@126.com (Y.L.); zzzjjx@outlook.com (J.Z.); gzb8607@163.com (Z.G.); zbdfst@yeah.net (B.Z.); 2 Engineering Research Centre of Fujian-Taiwan Special Marine Food Processing and Nutrition, Ministry of Education, Fuzhou 350002, China; 3 China-Ireland International Cooperation Center for Food Material Science and Structure Design, Fujian Agriculture and Forestry University, Fuzhou 350002, China; songmiao1969@yeah.net; 4 Institute of Food Science and Technology, Fujian Agriculture and Forestry University, 18 Simon Pit Road, Fuzhou 350002, China; 5 Fujian Provincial Key Laboratory of Quality Science and Processing Technology in Special Starch, Fujian Agriculture and Forestry University, Fuzhou 350002, China; 6 Teagasc Food Research Centre, Food Chemistry and Technology Department, Moorepark, Fermoy, Co. Cork, Ireland

**Keywords:** surimi, *Raphanus sativus*, vacuum frying, quality, nutrition, color, protein content, moisture

## Abstract

This study uses a response surface methodology to optimize the vacuum deep frying process of surimi cubes. The effects of vacuum deep frying temperature, frying time, and thickness on the hardness and color difference of surimi cubes with *Raphanus sativus* were studied. Further, the manuscript explored the quality changes of surimi cubes under different frying processes (vacuum deep frying, atmospheric deep frying, and shallow frying). Moreover, the Chinese Min-Cantonese cuisine-*Raphanus sativus* was utilized as auxiliary raw material to change the hardness and reduce the oil content. The optimal parameters of response surface methodology determined were: vacuum deep frying temperature 130 °C, frying time 900 s, and thickness 0.75 cm. Additionally, under this process condition, the hardness of the surimi chunks was 2015 ± 48.17 g, and the color difference was 23.27 ± 1.86. Surimi cubes without *Raphanus sativus* have superior elasticity and low hardness, and surimi cubes with *Raphanus sativus* have little color difference and high chewability. After the vacuum deep frying process, there was a high protein content and superior crispness. Shallow frying and adding *Raphanus sativus* effectively reduced the product’s oil content. Therefore, *Raphanus sativus* is suitable as a potential nutritional supplement to broaden its application in fried surimi foods.

## 1. Introduction

Historically, frying has been widely used as a common food processing method throughout the world as it can enhance the flavor of food. During the frying process, a heat transfer phenomenon occurs on the contact surface between food and oil through the pores on the food surface [1]. The phenomenon primarily occurs on the contact surface between the food and oil, which drains water and sucks oil continually through the pores on the surface of the food. Numerous studies have shown that including an excessive quantity of atmospherically fried foods in the diet leads to coronary heart disease. Moreover, consuming such foods over a long period may also be related to breast, colon, and prostate cancer [2]. Besides, high temperature can lead to the destruction of food nutrients and may also produce other toxic substances, such as acrylamide [3]. In addition, the oil used for cooking will be oxidized, hydrolyzed, and polymerized, which will lead to quality fission [4]. However, it is difficult for consumers to give up the fried foods. Therefore, a healthier frying process is urgently needed by the consumers and the food industry.

At present, some pretreatments have been adopted to improve the flavor and quality of food, such as blanching [5], hot air drying [6], and superheated steam drying [7]. Meanwhile, others have tried adding hydrophilic colloids to change the osmotic pressure of food [8], including base cellulose and hydroxypropyl methylcellulose to reduce oil absorption [4]. However, reducing the oil content can also lead to the loss of the original flavor, taste, and color of fried food.

Presently, vacuum deep frying is gaining popularity as a frying technology with several advantages. For instance, vacuum deep frying occurs at lower frying temperatures with lesser contact with oxygen [9]. Other verified advantages of vacuum deep frying include: (1) retaining the nutritional value of fried products; (2) reducing the oil content of fried foods; (3) protecting the natural color and flavor; and (4) reducing fat deterioration [10,11,12,13]. Moreover, vacuum deep frying is primarily used to process fruits and vegetables [14]. It also has the potential to be used as an alternative technology to produce healthy food and agricultural products, such as gilthead sea bream, potatoes, and bananas [15,16,17].

Surimi is a seafood product that uses minced fish as the primary raw material. Presently, the most common surimi products in the market include crab sticks, fish balls, fish cakes, fish tofu, and other frozen products. Comparing how conventional frying and vacuum deep frying affect the oil absorption and frying quality of fish tofu has revealed that vacuum deep frying reduces the final oil content of fish tofu more than conventional frying. Besides, there is minimal color change when vacuum frying was employed [18]. However, so far, few studies have compared the quality of surimi food under different frying processes.

Pastries with pieces of *Raphanus sativus* are a popular traditional local snack in Southern China. The primary raw materials of traditional *Raphanus sativus* include sticky rice noodles and shredded *Raphanus sativus*. Generally, the process of making the pastries include immersing and polishing the sticky rice before grating and cooking the radish and adding the condiments to mix the two, and steaming it [19]. *Raphanus sativus* increases the soft texture, water retention, and the product’s umami taste. Most families make these pastries during the Chinese New Year, which means a bumper harvest in the coming year. Therefore, it is popular among consumers in the Chinese market.

Thus, the optimal process parameters of the surimi cubes with added *Raphanus sativus* are determined by the response surface methodology. Moreover, how different frying technologies (vacuum deep frying, atmospheric deep frying, and shallow frying) affect the quality of surimi cubes to provide a theoretical basis for the research and development of surimi products were compared in this study.

## 2. Materials and Methods

### 2.1. Surimi Cubes Preparation

Golden thread surimi was purchased from the Food Co., Ltd. (Haixin Inc., Fuzhou, China). Eggs, *Raphanus sativus*, and garlic were obtained from the Superstore (YongHui Inc., Fujian, China). Bread crumbs and crispy fried flour were purchased from the Food Co., Ltd. (Jiaxian Inc., Chongqing, China). Carrageenan was purchased from a Bioengineering Co., Ltd. (Tengzhou Xiangning Inc., Shandong, China). Edible salt was obtained from the Salt Co., Ltd. (Zhongyan Inc., Shanghai, China). Peanut oil was purchased from the Grain and Oil Industry Co., Ltd. (Yijiang Inc., Jiangsu, China). Hanxing Haochi Sweet Fish Cake Bar was purchased from a Food Co., Ltd. (Hanxing Inc., Shanghai, China).

### 2.2. Frying Pretreatment

First, the frozen surimi was defrosted at 4 °C for 24 h. Afterward, the *Raphanus sativus* was peeled, weighed, and shredded before boiling for 5 min. Second, the surimi cubes with added *Raphanus sativus* were mixed with other ingredients (salt, minced garlic, carrageenan), refrigerated at −20 °C for 48 h. Third, the surimi cubes were cut into small pieces with a 4 cm long and wide mold. Next, surimi cubes were wrapped with egg white and coated with fried powder twice (in the same way as the first time), the surfaces of which were evenly sprinkled with bread crumbs. 

Each piece of surimi added with added *Raphanus sativus* in the experimental group contained surimi (dry weight 100 g), Raphanus sativus (50 g), salt (1.5 g), minced garlic (5 g), and 1.7 g of carrageenan (1.7 g). The control group (no *Raphanus sativus* group) contained surimi (150 g), salt (1.5 g), minced garlic (5 g), and carrageenan (1.7 g).

### 2.3. Frying Equipment

Each of the three frying processes requires different equipment. The vacuum deep frying is performed on a Vacuum Fryer (QS-VF05, Quanshi Food Machinery Co., Ltd., Shanghai, China).The feeding frame size is φ300 × 150 (mm), the operational vacuum degree is −0.092~−0.096, and the control temperature is 80–160 °C, the cylinder volume is 50 L, and the power is 19 KW. The atmospheric frying experiment is performed on a RJ-81D fryer (Xiangma Industrial Inc., Guangdong, China). The size of the feeding frame is 250 × 190 × 70 (mm), the control temperature is 60–200 °C, the volume of the cylinder is 10 L, and the power is 2.5 KW. The shallow frying experiment is performed on a JLW2601D pan (Midea Electric Inc., Guangdong, China), it has a volume of φ200 (bottom of the pan) ×48 (mm), and it is heated by a C2-2ST3304 embedded induction cooker (Midea Electric Inc., Guangdong, China) with 3.3 KW rated power.

### 2.4. Frying Conditions

The three types of frying equipment used different volumes of oil. Specifically, the vacuum deep fryer used 30 L of peanut oil, the atmospheric deep fryer used 8 L of the same type of peanut oil, and frying in the pan used 300 mL of the same type of peanut oil. The frying temperature was set as follows: 90 °C, 100 °C, 110 °C, 120 °C, 130 °C, 140 °C, and 150 °C, respectively. The frying time was 300 s, 450 s, 600 s, 750 s, and 900 s, respectively. The frying thickness was 0.5 mm, 0.75 mm, 1 mm, 1.25 mm, and 1.5 mm, respectively.

### 2.5. Vacuum Deep Frying Experiments

In order to avoid errors in the heating process, preheating is required in advance. Preheat to the setting temperature for 5 min each group before formal experiment to ensure that the temperature corrected. The surimi cube pieces (~25 g) are put into each round of experiment for automatic program. The indicators are measured when samples were cooled to room temperature.

### 2.6. Atmospheric Deep Frying Experiment

We performed a preheat to setting temperature for 5 min. The surimi cube pieces (~25 g) were loaded into the fryer baskets and then immersed into hot oil for any time. The grease on surface was dried with oil absorbent paper after experiments. The indicators were measured when cooled down to room temperature.

### 2.7. Shallow Frying Experiments

About 300 mL of oil was preheated to setting temperature in pan for 5 min. The surimi cube pieces (~25 g) were fried for any time. The samples were turned over every 60 s to avoid overheating. The grease on surface was dried with oil absorbent paper after the experiment. The indicators were measured when samples were cooled to room temperature.

### 2.8. Single Factor Experimental Design

The following single-factor experiments were carried out under a −0.095 MPA vacuum condition:

(1) Under different frying temperature conditions (110 °C, 115 °C, 120 °C, 125 °C, 130 °C, 135 °C, and 150 °C, respectively), the frying time was set to 1000 s, and the chip thickness was 0.75 cm to study how different temperatures affect the hardness, chewiness, and color difference of the surimi cubes with *Raphanus sativus*.

(2) Under different frying time conditions (800 s, 900 s, 1000 s, 1100 s, and 1200 s, respectively), the frying temperature was set to 125 °C, and the crisp slice thickness to 0.75 cm to study how different frying times affect the hardness, chewiness, and color difference of surimi cubes with *Raphanus sativus*.

(3) Under different thickness conditions (0.65 mm, 0.7 mm, 0.75 mm, 0.8 mm, and 0.85 mm, respectively), the frying temperature was set to 125 °C, and the frying time to 1000 s to study how different thicknesses affect the hardness, chewiness, and color difference of the surimi cubes with *Raphanus sativus*.

### 2.9. Response Surface Design

According to the results of the single-factor experiments, the independent variable ranges of the three factors of frying time (*A*), frying temperature (*B*), and thickness (*C*). The hardness (*R*_1_) and color difference (*R*_2_) of the vacuum deep fried surimi cubes with *Raphanus sativus* serve as response values were determined. These parameters helped determine and verify the optimum extraction conditions for vacuum deep frying the surimi cubes with *Raphanus sativus*. Table 1 shows the response surface design factor levels and codes.

### 2.10. Texture Analysis

A modified version of the test method is adopted in Yu et al. [20]. In this experiment, the hardness is used as the characteristic index in the single factor test and response surface test, and hardness, springiness, chewiness, and cohesiveness are used to study effects of different processes on the texture properties of the surimi cubes. A TA-XT_2_ texture analyzer (Stable Micro System Inc., Godalming, UK) was used to determine index, compression mode test is selected, and the parameters were set as follows: 1 mm/sec pre-test speed, 1 mm/sec mid-test speed, 0.5 mm/sec post-test speed, 30 mm initial test height, and a 5 g trigger force. The compression thickness ratio was set to 75%. The test was repeated with ten pieces of the surimi cubes with *Raphanus sativus* to obtain the average value.

### 2.11. Color Values

An CS-10 automatic colorimeter (Caipu Technology Inc., Hangzhou, China) was used to detect the color of the surimi cubes added with *Raphanus sativus*. The operational parameters were as follows: A 10-mm diameter for the color measurement spot and a standard whiteboard was used as the standard sample. A Hunter uniform color system was used to measure L*, a*, b*, and ΔE*. Where L* represents brightness, a* value represents red (+)/green (−), b* value corresponds to yellow (+)/blue (−), and ΔE* denotes the color difference.

One side of the sample was selected randomly to test some areas of the side using a color difference meter to obtain the L* value, a* value, and b* value, respectively. The color of the Hanxing Haochi Sweet Fish Cake Bar was used as the standard to obtain *L*_0_* = 61.91, *a*_0_ = −138.97, and *b*_0_ = 49.36. Using the obtained values, the color difference Δ*E* of the surimi cubes with added *Raphanus sativus* was calculated using Formula (1). The experiment was repeated three times to obtain the average value for later use.

(1) $$\Delta E=\sqrt{{\left(L_{0}*-L*\right)}^{2}+{\left(a_{0}*-a*\right)}^{2}+{\left(b_{0}*-b*\right)}^{2}}$$

In Formula (1) shown above, L_0_* denotes the brightness value of the Hanxing Haochi Sweet Fish Cake Bar, *a_0_* corresponds to the redness value of the Korean star delicious sweet fish cake stick, *b_0_* represents the yellowness value of the Korean star delicious sweet fish cake stick, L* signifies the measured brightness value, a* corresponds to the measured redness value, and b* represents the measured yellowness value.

### 2.12. Determination of Water Content

The Chinese national standard GB 5009.3-2016 “Determination of Moisture in Food” is referred when doing the analysis. Accordingly, the samples were added to the SFY-20A quick moisture apparatus (Guanya Electronic Technology Inc., Shenzhen, China), dried to constant weight, then the moisture content was displayed on the detector through gravimetric method. The measurements were taken thrice in parallel, and the average value was obtained.

### 2.13. Determination of Protein Content

The total protein content was determined by the UDK 159 classical Kjeldahl method (VELP Scientific Inc., Usmate, Italy). The surimi cubes with *Raphanus sativus* were considered after various frying processes at 130 °C for 900 s; 200 mg of samples, 2 Kjeltabs catalyst, and 12 mL of 98% H_2_SO_4_ were added into 250 mL digestion tubes and digested by FOSS Tecator Digestion System 8 (Foss Analytical Inc., Sweden) at 420 °C for 60 min. The samples were titrated using 0.1 N HCl and the crude protein content was calculated with a conversion factor of 6.25. 

### 2.14. Determination of fat Content

The surimi cubes with *Raphanus sativus* were considered after various frying processes at 130 °C for 900 s. The fat content of surimi cubes with *Raphanus sativus* was determined by automatic SZF-06 Soxhlet solvent extractor (Jiading Food and Oil Instrument Inc., Shanghai, China). The surimi cubes with *Raphanus sativus* under the same treatment conditions were measured in parallel three times, then the average value was taken.

### 2.15. Statistical Analysis

All experimental data were repeated 3 times, and the average value was taken. Design Expert.V8.0.6 statistical software (Stat-Ease Inc., Minneapolis, MN, USA) was used for experimental design and data analysis. All statistical analyses were performed using DPS V9.05 Data Processing System software (Science Press Inc., Beijing, China). ANOVA was used for analysis of variance, and Duncan’s test was used for significance analysis; *p* > 0.05 indicates that the difference is not significant, and *p* < 0.05 indicates that the difference is significant.

## 3. Results and Discussion

### 3.1. The Influence of Different Factors on the Texture Characteristics of Surimi Cubes 

**Figure 1:**
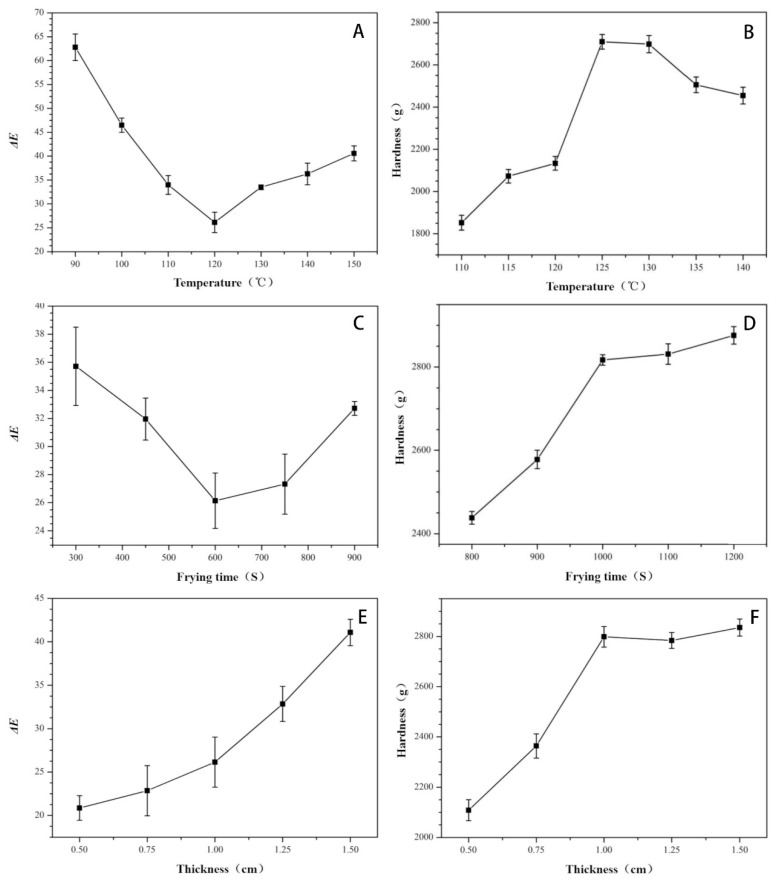
Effect of different factors on the textural quality of the *Raphanus sativus*-added surimi cubes ((**A**) the influence of temperature on Δ*E*, (**B**) the influence of temperature on hardness, (**C**) the influence of frying time on Δ*E*; (**D**) the influence of frying time on hardness; (**E**) the influence of thickness on Δ*E*; and (**F**) the influence of thickness on hardness).

From Figure 1A, it is evident that as the frying temperature increases, the Δ*E* value initially decreases before increasing. The color difference change could be attributed to the non-enzymatic browning and caramelization during the frying process [15]. Reportedly, the *L* value of the apple slices decrease as the frying temperature increases [21], which gives the product a better color. However, as the frying temperature increases, the *L* value reduces considerably, and the color difference with the standard product increases, exhibiting a V-shape. It could be due to an increasing in a* value, which related to the presence of carotenoids. At higher temperatures (shorter frying times), higher degradation of compounds was observed [22].

In Figure 1B, as the frying temperature increases, the hardness of the surimi cubes with *Raphanus sativus* also increases. The higher hardness could be attributed to the high temperature of the fat and a decent heat transfer effect. Specifically, the heat denatures the fish protein, in turn, the muscle fibers toughen, and the structure becomes firmer. Simultaneously, the surface starts losing water rapidly, thereby forming a hard shell that increases the hardness. However, the internal moisture is not completely removed, which prevents the formation of a completely dense structure. Another results also showed that the hardness of air-fried surimi was further increased with the rise of air-frying times and temperatures [23]. Hence, the hardness is low, and the crispness is better. Besides, in low-temperature frying, the surface of the surimi cubes with *Raphanus sativus* loses water faster, resulting in wrinkles on the surface and reducing the dehydration rate by limiting the dehydration to within the cubes. Meanwhile, once the temperature of the oil reaches the boiling point, the water forms bubbles and bursts within the cubes. Similar studies have reported that after vacuum deep frying at 110 °C for 25 min, the breaking force of apple slices is less than 500 g, which has good crispness [21].

Figure 1C shows a similar trend in the extension of the frying time. Accordingly, once the frying time reaches 1000 s, the surimi cubes with *Raphanus sativus* have fully undergone the Maillard reaction, lipid oxidation and caramelization reaction, making the surface golden yellow and the shell crispy. Later, the *L* value reduces due to prolonged frying time, and the color difference value gradually increases. The change in color is more different and prominent than the standard color. However, at this point, it is most likely that the fish filet would burn. Therefore, it is crucial to control the frying time.

According to Figure 1D, with the extension of the frying time, the hardness of the surimi cubes with *Raphanus sativus* also increases. Once the frying time reaches 1000 s, the hardness value is almost flat. When the frying time gradually increases, the myofibrillar protein will expand and dissociate under high-temperature conditions, forming more polymerized disulfide bonds that will condense and contract the myofibrillar protein. Ultimately, it will lead to an increase in hardness [24]. Currently, as the frying time is prolonged, the salt-soluble protein would dissolve and combine with water under a low-pressure environment. In other words, the surimi cubes with *Raphanus sativus* reach a dehydrated state quickly and forms a hard shell on the surface. Therefore, a stable structure is formed following a short time of frying [10].

In Figure 1E, a greater thickness is accompanied by a higher Δ*E*. It could be due to the oxidative denaturation of myoglobin under high-temperature conditions, which forms metmyoglobin. The cellular structures of meat could be damaged by heating, which lead to more exposed oxygen. Lipid and protein molecules were attacked subsequently by the triggers of reactive oxygen species [25]. *Surimi cubes* with *Raphanus sativus* form metmyoglobin at a slower rate due to the large thickness. As a result, the shell color does not become golden yellow. Meanwhile, since the shell quickly forms a hard shell under low-pressure conditions, the water content cubed into surimi cubes with a large thickness, and the presence of water makes the color lighter.

As Figure 1F indicates, as the thickness increases, the hardness of the surimi cubes with *Raphanus sativus* first increases and then tends to level off. It is most likely because the thin *Raphanus sativus*-added surimi cubes dehydrate quickly and forms a hard shell. Once the thickness increases to a certain degree, the myofibril protein network structure within the *Raphanus sativus*-added surimi cubes becomes denser and thicker. However, the internal moisture is not removed at the same time and temperature. Starting from a 0.75-cm thickness, the hardness value increases slowly.

The surface of the *Raphanus sativus*-added surimi cubes is golden yellow with a crispy shell. The change in color difference primarily originates from the Maillard reaction of starch and protein during the frying process. As a result, a* (redness) and b* (yellowness) increase, giving the product a more attractive color. Besides, the *L* value of the *Raphanus sativus*-added surimi cubes increases as the frying temperature or frying time increases. In turn, the Δ*E* of the standard product increases, exhibiting a V-shape.

The frying temperature reaches 125 °C when the frying time is 1000 s, and the thickness reaches 0.75 cm. At this point, the hardness of the *Raphanus sativus*-added surimi cubes is close to the maximum value, and the Δ*E* value is closest to the standard sample, with attractive color and the least processing time.

### 3.2. Optimal Parameter Optimization of Response Surface

According to single-factor test results, the chewiness and hardness basically exhibited an identical trend. Therefore, the response surface design only considers the hardness and color difference as response values. Table 2 lists the experimental design and results of the response surface analysis of the surimi cubes with *Raphanus sativus* in vacuum deep frying. The Design Expert.V8.0.6 statistical software was used to perform multiple regression fitting on the experimental results.

In the regression equation model, *p* < 0.0001 indicates extreme significance (Table 3). The lack of fit term p of hardness is 0.8631, and the lack of fit term of chromatic aberration is 0.385. Hence, both are greater than 0.05. Meanwhile, the hardness corresponding to *R*_2_ and *R*_2adj_ are 0.9997 and 0.9991, respectively. The color difference corresponding to *R*_2_ and *R*_2adj_ are 0.9932 and 0.9810, respectively. According to these results, the equation has good simulation properties. It is suitable to optimize the process parameters in the vacuum deep frying process of the *Raphanus sativus*-added surimi cubes. The order of the influence of each factor on the hardness response value is as follows: frying time > frying temperature > thickness; the order of the influence of each factor on the corresponding value of the color difference is: frying temperature > frying time > thickness.

The Design-Expert.V8.0.6 software was used to obtain the second-order multivariate polynomial regression of the frying time, temperature, and thickness on the hardness and color difference of the *Raphanus sativus*-added surimi cubes.

*R*_1_ = 10,464.73 + 3901.57*A* + 546.47*B* + 244.24*C* + 1352.88*AB* + 31,745*AC* − 987.85*BC* + 3606.17 *A*^2^ − 5642.94*B*^2^ − 608.3*C*^2^

*R*_2_ = 25.11 + 0.68*A* + 0.87*B* + 0.62*C* + 2.48*AB* + 0.11*AC* − 1.8*BC* − 3.12*A*^2^+ 3.89*B*^2^ + 0.46*C*^2^

In the formula: *R*_1_—hardness, g; *R*_2_—color difference; *A*—frying time, s; *B*—frying temperature, °C; *C*—thickness, cm.

Furthermore, the regression equation model has *p* < 0.001 in Table 3, which shows extreme significance. In regards to the linear effect of the indicators, the hardness and color difference indicators corresponding to the frying time, frying temperature, and thickness exhibit extreme significance (*p* < 0.01). In contrast with the two indicators of hardness and color difference, the effects of frying time, temperature, and thickness on hardness have similar outcomes. Relative to the color difference, the thickness has less influence on the hardness than the frying time and temperature. In analyzing the different interactions, the interaction of the frying time and temperature has a significant impact on the hardness and color difference (*p* < 0.01). Moreover, the interaction of the frying time and thickness has a significant impact on the hardness (*p* < 0.01). Additionally, it also has a significant impact on the color difference. The effect is insignificant (*p* > 0.05). The interactive effects of frying temperature and thickness have extremely significant effects on the hardness and color difference (*p* < 0.01). Out of the effects of the quadratic term, frying time and temperature have an extremely significant impact on the hardness and color difference (*p* < 0.01). Meanwhile, the thickness also has a significant effect on the hardness (*p* < 0.01). However, it has no significant effect on the color difference (*p* > 0.05).

### 3.3. Response Surface Analysis Results

**Figure 2:**
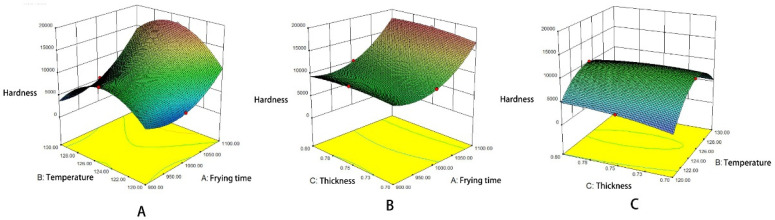
Response surface plots of interaction on the hardness (*R*_1_). (**A**) Temperature-Frying time, (**B**) Thickness-Frying time, (**C**) Thickness- Temperature.

A three-dimensional two-factor interaction diagram of the regression equation was obtained based on the response value obtained from the interaction above. Figure 2 illustrates the influence trend of the frying time, frying temperature, and thickness on the hardness of the *Raphanus sativus*-added surimi cubes. According to the diagram, the interaction of the three factors has a significant influence on the hardness. Besides, the three-dimensional curve shows a significant change. As shown in Figure 2A, the hardness initially increases before decreasing as the temperature starts to rise. Moreover, the same curve also shows how the hardness increases gradually with time. According to Figure 2B, the response surface of the thickness and time are relatively flat, implying that the surface thickness has a relatively smaller influence on the hardness. On the other hand, it is evident that frying time has a more obvious effect on hardness. According to Figure 2C, the hardness remains largely constant as the thickness increases. However, as the frying temperature increases, the hardness first increases before decreasing. Overall, the frying time and frying temperature have a distinct impact on the hardness response value. As a result, the curved surface is steeper. In addition, the result is consistent with those from the variance analysis of the regression equation (the effect of thickness is negligible). It indicates that the response surface optimization design can better reflect how the frying time, temperature, and thickness influence the hardness of the *Raphanus sativus*-added surimi cubes. It has been reported that at a certain frying temperature, the surface of the material forms a hard shell. At this point, the internal structure forms a dense structure. As a result, the thickness change has no effect on the value of the hardness.

**Figure 3:**
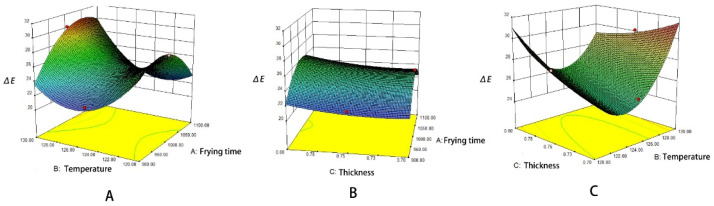
Response surface plots of interaction on color (*R*_2_). (**A**) Temperature-Frying time, (**B**) Thickness-Frying time, (**C**) Thickness- Temperature.

Figure 3 shows the influence trend of frying time, frying temperature, and thickness on the *Raphanus sativus*-added surimi cubes. Accordingly, the interaction of the three factors has a significant influence on the chromatic aberration, which is further proved by the drastic changes in the three-dimensional curves. According to Figure 3A, as the temperature increases, the color difference first decreases before increasing, and as time goes by, it keeps increasing and then decreases. The heat and mass transfer cause the color change in the surimi, leading to a loss of water and an increase in the oil content of the surimi. As a result, the initial rise of the curve and the decrease that follows could be attributed to the dehydration process during the first half of frying. Meanwhile, the second half of the frying process is primarily governed by oil absorption and the main Maillard reaction. Therefore, the color difference generates identical changes [26]. The outcome is similar to the results of the significance analysis. The frying time and temperature inflict a significant level of color difference (*p* < 0.01). As illustrated in Figure 3B, the change in thickness only has a slight effect on the chromatic aberration, and as time goes by, the chromatic aberration first increases before decreasing. The overall curve is relatively flat, which is similar to the results from the significance analysis, and the effect is less significant (*p* > 0.05). According to Figure 3C, the color difference decreases slightly as the thickness increases. However, as the frying temperature increases, the color difference initially exhibits a decreasing trend before increasing. Overall, frying temperature and time are the main influential factors that affect the color difference of the *Raphanus sativus*-added surimi cubes, and the underlying reason is the Maillard reaction, which changes the color of food at high temperatures.

The Design Expert.V8.0.6 software was used to analyze all experimental data. The largest hardness and the smallest color difference for the *Raphanus sativus*-added surimi cubes was obtained using the software. Accordingly, the following parameters were obtained: frying temperature was 125.97 °C, frying time was 900.14 s, and the thickness was 0.75 cm, and the model predicted the hardness and color difference to be 1988 g and 21.1436, respectively.

During the actual frying process, there is a high likelihood to produce acrylamide if the frying temperature is too high [3]. The material and an extended frying time increase the cost of extra time. Therefore, the vacuum deep frying temperature was determined as 125.00 °C after correction for the actual situation, the corresponding frying time is 900 s, and the chip thickness is 0.75 cm.

The experiments were repeated to verify the accuracy of the model through the best process parameters and the results are listed in Table 4. All parameters and indicators conform to the theoretically predicted values. Therefore, it can be stated that the regression model is reliable. When the vacuum deep frying experiments were repeated at 125.00 °C for 900 s, and 0.75-cm thickness, the average values of the test results of each index were as follows: the hardness of the *Raphanus sativus*-added surimi cubes was 2015 ± 48.17 g and the color difference was 23.27 ± 1.86, which is consistent with the theoretically predicted value. Overall, the regression model is reliable.

### 3.4. Effects of Different Processes on the Texture Properties of the Surimi Cubes

**Figure 4:**
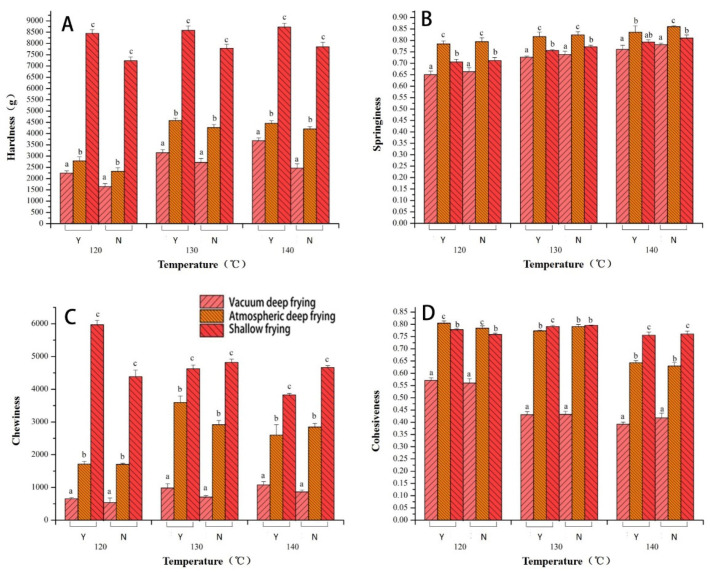
Effect of different processes on the texture of surimi cubes ((**A**) the influence of temperature on hardness, (**B**) the influence of temperature on springiness, (**C**) the influence of temperature on chewiness, (**D**) the influence of temperature on cohesiveness. Y: *Raphanus sativus* added; N: no *Raphanus sativus*). Different letters in the same chart represent significant differences between different treatments according to Duncan’s multiple range test. (*p* < 0.05).

Hardness is described as the compactness of structural organization for starch and protein complexes. In Figure 4A, the hardness of the vacuum deep fried surimi cubes increases as the temperature increases. In contrast, the hardness of the atmospherically deep fried surimi cubes first increases and then stabilizes as the temperature increases. Reportedly, higher frying temperatures cause the surface of the sample to form a harder crust much faster, resulting in a greater hardness [27]. When experimented at a single temperature, the order of hardness value from high to low is as follows: shallow frying > atmospheric deep frying > vacuum deep frying. The outcome could be attributed to a lower maximum breaking force value under vacuum conditions [10]. Under vacuum conditions, vacuum deep frying tends to lose water quickly, which forms large and dense pores, giving a crispy texture to the skin, while the internal surimi contains a lower hardness. The increasing hardness of crackers could be due to higher loss of moisture content. Thus, the higher water loss produces harder and drier texture [28]. Under the decoction process, the hardness is higher due to lesser dehydration, resulting in the retaining of additional liquid (water, oil) within the sample. Consequently, the density increases, which in turn, increases the hardness. The loss of water during the frying process is the primary reason for the textural change [11]. Dehydration forms a harder shell, which increases the sample’s crispness, and its hardness value is relatively low. Thus, the hardness value of the *Raphanus sativus*-added surimi cubes primarily depends on the moisture content [29]. When comparing the change in hardness value with or without adding *Raphanus sativus*, it was found that at any temperature, the hardness value of the *Raphanus sativus*-added surimi cubes was greater than that of surimi cubes without *Raphanus sativus*. It could be explained by the higher water content in *Raphanus sativus*, which has the characteristics of dietary fiber. Thus, when the oil penetrates the sample during the frying process, the surimi cubes and the surface of the *Raphanus sativus* lose water simultaneously, resulting in a relatively higher hardness value. It was found that the possible reason is more uniform porosity was observed on the vertical cross-section of carrot chips which change the quality of carrot [13].

In Figure 4B, the elasticity of the *Raphanus sativus*-added surimi cubes increases when the temperature of the three frying processes increases. Among them, the elasticity value in descending order is as follows: atmospheric deep frying > shallow frying > vacuum deep frying. Noticeably, the vacuum deep fried samples have the least elastic value. It has been reported that the largest difference between vacuum deep frying and atmospheric deep frying lies in the structural changes on the surface, which include smaller puffing and smaller pores. The atmospheric deep frying has a relatively larger volume expansion with larger pores, which is more elastic [11].

Furthermore, the production process of the *Raphanus sativus*-added surimi cubes involves dipping the frying powder on the surface, which results in the starch gelatinization phenomenon on the surface. For the non-vacuum deep frying processes, the hard shell formed by the gelatinization of starch on the surface hinders the discharge of internal bubbles. Consequently, the process intensifies the volume expansion, which increases the elasticity [30]. The surimi cubes processed by vacuum deep frying benefit from lower water vapor pressure. More specifically, they have less volume expansion, resulting in weaker elasticity. However, since the frying process heats one side at a time, there is a higher chance of uneven heat dissipation, resulting in disordering the internal structure of the *Raphanus sativus*- added surimi cubes, which reduces the elasticity. Besides, the elasticity of the *Raphanus sativus*-added surimi cubes was slightly smaller than that of the surimi cubes without *Raphanus sativus*. The reason may be that the surimi cubes without radish could form a stronger and denser surimi gel during the frying process.

According to Figure 4C, chewiness is an indicator of the energy required to break the semi-solid sample into a stable state when swallowed. The chewiness rated in a descending order is as follows: shallow frying > atmospheric deep frying > vacuum deep frying. The order is similar to the changing trend of hardness. Generally, chewiness is not proportional to crispness. In other words, a stronger chewiness corresponds to inferior crispness. Thus, the higher hardness and moisture content in the decoction process produces higher chewiness. However, in atmospheric deep frying and vacuum deep frying, the sample is completely immersed in oil for frying and dehydration. Therefore, the moisture content is low. It results in a crispy mouthfeel, and there is a significant difference in chewiness (*p* < 0.05). From the analysis of the source of texture chewiness, chewiness is equal to hardness × elasticity × cohesion. Therefore, the changing trend is similar to that of hardness. Reportedly, a microstructure analysis was carried out showed that after vacuum deep frying, the sample has a finer and denser pore structure, and there will be wrinkles on the surface due to rapid water loss. Such structural features affect the overall chewiness of the sample [31]. Indeed, atmospheric deep frying produces relatively large pores, resulting in higher chewiness. Moreover, the chewiness of *Raphanus sativus*-added surimi cubes was higher than the surimi cubes without *Raphanus sativus.* The most applicable explanation is that the water content in *Raphanus sativus* makes the inside of the sample have a higher water content. It produces a juicy taste and improves chewiness.

Cohesiveness corresponds to the relative work produced by compressing the sample twice in the TPA mode of the Texture Analyzer. More specifically, the cohesiveness is used to express the change between two deformations of the *Raphanus sativus*-added surimi cubes. According to Figure 4D, the cohesiveness tends to decrease as the temperature increases. In particular, the decrease in vacuum deep frying is obvious, and the cohesiveness associated with the vacuum deep frying process is significantly lower than the other two processes (*p* < 0.05). As mentioned above, the *Raphanus sativus*-added surimi cubes have a lower moisture content after vacuum deep frying, which could be due to the uniform and uneven thermal denaturation of myosin and actin [32]. As a result, the skin forms a hard shell. After compressing the *Raphanus sativus*-added surimi cubes for the first time, the shell was damaged from the compression. So, the damaged sample could not produce the same resistance as the first compression during the second compression. Therefore, there is a relatively large work difference between the two compressions. Hence, the cohesion is low. It may be because the food crust microstructure affects the oil absorption rate, which changes the quality [33]. When frying at low temperatures, the cohesiveness of atmospheric deep frying is higher than shallow frying. However, the result is the opposite at higher temperatures. This could be attributed to the retained water content in *Raphanus sativus*-added surimi cubes when frying at lower temperatures [34]. Besides, the shell starch has not been fully gelatinized to form a hard shell, so the original shape can be better maintained during the two compressions. At 120 °C, atmospheric deep frying has higher cohesiveness than shallow frying. As the temperature of the frying process increases, the atmospherically fried sample’s water content diminishes, and the volume expands steadily to form a crispy crust close to vacuum deep frying. Under further compression, the crust becomes damaged, and the cohesiveness decreases. In contrast, the shallow frying process retains a higher water content than atmospheric deep frying, which results in higher cohesiveness. Importantly, the presence or absence of *Raphanus sativus* has no obvious effect on the cohesiveness of the surimi cubes.

### 3.5. The Effect of Different Frying Techniques on the Color Difference of Surimi Cubes 

**Figure 5:**
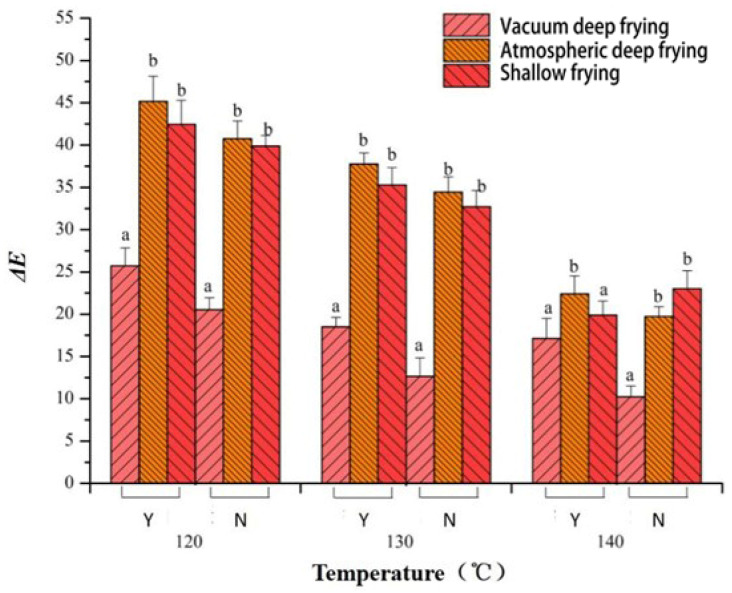
Effect of different temperatures on color difference of surimi cubes with different frying technologies (Y: radish added; N: no radish). Different letters in the same chart represent significant differences between different treatments according to Duncan’s multiple range test. (*p* < 0.05).

**Figure 6:**
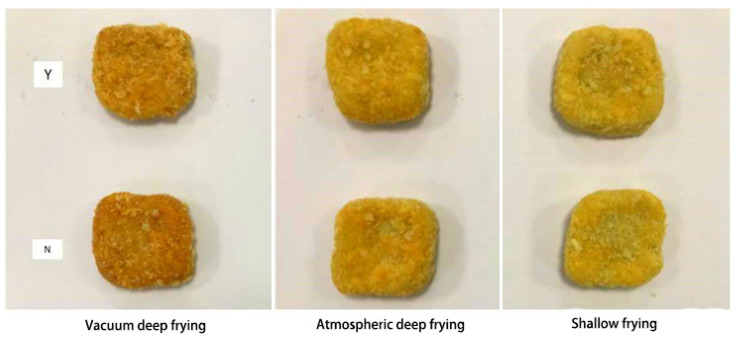
Effect of different frying processes on surimi cubes (Y: *Raphanus sativus* added; N: no *Raphanus sativus*).

In Figure 5, the Δ*E* value of the surimi cubes with added *Raphanus sativus* tends to decrease as the frying temperature increases. The Δ*E* value of the surimi cubes was lowest after vacuum deep frying, followed by the shallow fried surimi cubes, and the Δ*E* of atmospherically deep fried surimi cubes was the highest. The result indicates that vacuum deep frying has a higher capacity to retain the original color of the sample and reduce color deterioration. The best explanation is that at lower temperatures, the rate of chemical reactions is reduced, such as ascorbic acid oxidation, making the color difference less prominent. The vacuum fried squid has lower L* value than atmospheric frying, which is mainly caused by Maillard reaction during heating [35]. In addition, light reflection was potentially decreased by oil absorption, which lead to the increasing of L* value [36]. Moreover, the color of the standard used in this experiment is dark and slightly reddish. From Figure 6, the surface of the *Raphanus sativus*-added surimi cubes after vacuum deep frying is brownish red, the atmospherically deep fried sample is yellow, and the shallow fried sample exhibits a golden color. When compared with the reference product of the experiment, after vacuum deep frying, the surface of the surimi cubes with *Raphanus sativus* exhibited a darker and reddish color, and the color is much closer to the reference product. This phenomenon is related to non-enzymatic browning. However, this result contradicts the outcomes of [37], and the possible reason is that since it is difficult to accurately control the heating time in the vacuum deep frying process, a time error exists in the experiment. Generally, the frying time can significantly affect the sample’s color difference, which leads to the results of this experiment [21].

Furthermore, the *Raphanus sativus*-added surimi cubes had a lower Δ*E* than the surimi cubes without *Raphanus sativus*, which could be explained by the difference in the water content of the two samples. The water distribution in the sample affects the production of non-enzymatic browning of the epidermis, and enzymatic browning is also relatively reduced when the water content is higher [38]. Besides, the *Raphanus sativus*-added surimi cubes can further diffuse the water in the shredded *Raphanus sativus* during the frying process, resulting in different Δ*E* values.

### 3.6. The Effect of Different Frying Techniques on the Moisture Content of Surimi Cubes 

**Figure 7:**
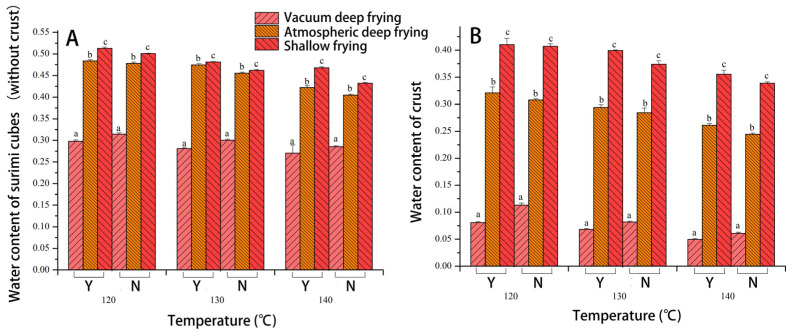
Effect of different frying techniques on water content of surimi cubes ((**A**) surimi cubes without crust; (**B**) crust; Y: *Raphanus sativus* added; N: no *Raphanus sativus*). Different letters in the same chart represent significant differences between different treatments according to Duncan’s multiple range test. (*p* < 0.05).

In order to detect the moisture content, the surimi cubes with *Raphanus sativus* were divided into two sections as inner and outer skin. Figure 7 shows how different frying temperatures and frying process conditions affect the moisture content of the inside and outer skin of the surimi cubes. When the frying temperature increases, the moisture content of the outer skin of the surimi cubes with *Raphanus sativus* exhibits a downward trend in Figure 7A. The moisture content of the three frying processes in descending order is as follows: shallow frying > atmospheric deep frying > vacuum deep frying. Since *Raphanus sativus* is not present in the skin component, the water content in the skins of the surimi cubes was not significantly different from surimi cubes without *Raphanus sativus* (*p* > 0.05). Meanwhile, after vacuum deep frying, the skin of surimi cubes contain less water. It could be related to the loss of water during the frying process that primarily occurs on the sample’s surface [38]. Besides, the vacuum environment may have caused the sample’s surface to undergo microstructural changes, and these changes could be different from the surface of the atmospherically fried sample, which, in turn, could reduce the internal water loss of the surimi cubes. Similar changes on surimi surface were happened with increasing of temperatures immediately when the hot air started to blow, which could reach boiling point more quickly and lead to a dried layer, preventing further loss of internal moisture during frying [39].

Fundamentally, the changing trend is the same as that of the outer skin. Meanwhile, according to Figure 7B, the water content of the surimi cubes with *Raphanus sativus* is higher than that of the outer skin. Consistent with the loss rate result, vacuum deep frying loss rate is the highest, and the moisture content is the least. The loss rate is least in the shallow fried samples, yet, the moisture content is the highest. It could be related to a higher oil absorption rate [37]. At the start of the frying process, the outer surface of the product is dry, and the internal moisture is converted into water vapor that dissipates, which generates a certain pressure gradient. As the frying time increases, after drying, the surface becomes more hydrophobic, allowing the sample to absorb more oil, leading to a reduction in moisture content [29]. Moreover, after vacuum deep frying is complete, the surface oil enters the product through capillary absorption when the system is vacuumed to restore the system to atmospheric pressure, resulting in higher oil absorption and lower moisture content [40]. At this stage, the oil absorption mechanism is different from that of atmospheric deep frying and shallow frying, primarily because atmospheric deep frying and shallow frying only absorb most of the oil during the cooling period after frying, resulting in reduced moisture [41]. The mechanical energy and viscosity were improved by loss of moisture which was related to the hardness, resulting in molecular stretching and dissociation [42]. When comparing the effects of adding *Raphanus sativus* to the surimi cubes, it was discovered that the surimi cubes without *Raphanus sativus* has a higher moisture content. This result is consistent with the loss rate trend. More specifically, the surimi cubes with *Raphanus sativus* have less moisture after vacuum deep frying. However, atmospheric deep frying and shallow frying are the opposite.

When comparing the difference in moisture content between crus meats, it was found that under the three frying processes, the moisture content of the outer skin is less than that of the inner meat. In combination with the results of the textural analysis, the outer skin had less moisture whilst the inner skin had more moisture, which is conducive for a crispy outside and tender inside. The primary reason for the difference lies in the changes in the microstructure of the oil contact surface. These include the formation of hard skin, cell wall shrinkage, cell separation, and other phenomena during dehydration. Collectively, these can reduce the ability of water migration [38,43]. Moreover, the decreasing of water content also made lower light refraction, which has positive correlation on Δ*E* [42]. Besides, frying at a lower temperature slows the diffusion of water [34]. The change law of the moisture content confirms the test results from Section 3.4, where the hardness value of the surimi cubes with *Raphanus sativus* is related to the moisture content. Specifically, a higher moisture content translates into greater hardness.

### 3.7. The Effect of Different Frying Techniques on Protein and Oil Content of Surimi Cubes 

As shown in Table 5, under different frying conditions, the protein content of the surimi cubes with *Raphanus sativus* has a significant difference (*p* < 0.05). The protein content in the surimi cubes with *Raphanus sativus* is sorted in descending order: vacuum deep frying > shallow frying > atmospheric deep frying, the oil content is shown in descending order: vacuum deep frying > atmospheric deep frying > shallow frying. Finally, the surimi cubes without *Raphanus sativus* have a relatively higher oil content than the surimi cubes with *Raphanus sativus.*

Relative to atmospheric deep frying and shallow frying, vacuum deep fried surimi cubes with *Raphanus sativus* have a higher protein content. This could be due to the lower temperature of vacuum deep frying, which reduces the deterioration of nutrients. Additionally, the low oxygen content in a vacuum environment also reduces deterioration through oxidation. Paulo et al. have found that vacuum deep frying can better retain the nutrients in plants: for example, the carotenoid content in vacuum fried mangoes, mung beans, and sweet potatoes is higher than that in atmospheric fried products which about 20–50% more [38], where vacuum deep frying better retained the nutrients in plants. Vacuum frying reduced the moisture and fat contents and maintained protein content than atmospheric frying [35]. Therefore, surimi cubes with *Raphanus sativus* contained higher protein content under the experimental conditions with lower oxygen content. However, only one side is in contact with the edible oil during the frying process, and the sides are turned over regularly, which results in uneven heat distribution and less protein loss than atmospheric deep frying. However, the thermal processing could generate a large amount of free radical production, which oxidized protein and lipid [44,45]. Lipid oxidation and hydrolysis, taking place during the course of deep-fat frying which could influence the quality attributes of the final product related to texture, flavor, and nutrient composition [46].

Furthermore, after vacuum deep frying, the oil content in the surimi cubes with *Raphanus sativus* was significantly higher than that of atmospheric deep frying and shallow frying. There are several possible reasons for this result: (1) The oil content increases with a longer frying time [13]. In the vacuum fryer used in the experiment, the oil was added to the frying area before the temperature of the oil reached the set temperature. Therefore, the actual frying time (calculated from the beginning of contact with oil) is longer than the defined frying time (750 s), resulting in higher oil content than atmospheric deep frying. A similar study showed that the absorption of frying oil took placed in clam tissue with lipid content increased more than two-fold [47]. (2) For a long time, it has been believed that the oil absorption phenomenon is related to the oil contact surface, which primarily occurs during the cooling process of the food leaving the oil bath after being fried, and the oil or water is sucked and discharged through the pores formed on the surface [31,48]. The association between oil content and water loss is very close, primarily because the number of holes for water evaporation determines the quantity of grease that can be sucked in. Fried fish crackers with a higher degree of expansion will form more gas and thus be able to capture more oil [49]. In addition, hydrophilic groups could be hydrogen-bonded with water molecules, resulted in the increased moisture content of sample crust [50]. Therefore, regardless of whether frying under vacuum or atmospheric conditions, the oil content is related to the pores and hydrophilic groups in the sample. The starch on the surface shell is not completely gelatinized under vacuum deep frying conditions, causing more fat to be sucked into the surimi cubes with *Raphanus sativus.* Akinpelu et al. found that vacuum fried plantain chips had more acceptable sensory properties, which related to gelatinization [10]. (3) It may also be related to the fact that the surface of the surimi cubes contains egg white. The crispy skin layer was not formed in the early stage of frying, allowing the water to be discharged quickly from the body. As the frying time is extended, a crispy skin layer is gradually formed, which locks the fat, forming a higher oil content. It is showed that the negative correlation between moisture and oil content. Water loss is maybe related to different vapor pressures, which force oil to enter the void left by evaporation of water [51].

## 4. Conclusions

A single-factor experiment in response to the vacuum deep frying process to optimize the parameters of surimi cubes with *Raphanus sativus* was combined in this study. Moreover, the quality of the surimi cubes (with or without *Raphanus sativus*) was also analyzed. In general, the vacuum deep fried surimi cubes with *Raphanus sativus* had the smallest hardness, low chewiness, and low cohesiveness. Besides, the vacuum deep fried surimi cubes also had good crispness. The hardness and chewiness of the atmospheric deep fried surimi cubes were slightly higher than those that were vacuum deep fried, and also had the highest elasticity and relatively high cohesion. The hardness and chewiness of the shallow deep fried surimi cubes were much higher than those of atmospheric deep frying, and the chewiness was strong. The surimi cubes with *Raphanus sativus* had higher hardness and chewiness than the surimi cubes without *Raphanus sativus.* In addition, the moisture content of the three frying processes in descending order was as follows: shallow frying > atmospheric deep frying > vacuum deep frying. Besides, the protein content of the surimi cubes in descending order was as follows: vacuum deep frying > shallow frying > conventional deep frying. The present research provided helpful insights for further exploration of the development of surimi food after vacuum deep frying, atmospheric deep frying, and shallow frying. However, when utilizing a vacuum deep frying process, the vacuum degree of the vacuum deep frying equipment is closely related to the product quality. The limited conditions in this experiment could be considered as a research index for further research when the conditions are available.

## Figures and Tables

**Table 1:** Factors and their coded levels used in experimental design for RSM.

Dependent Variable	Coded Value		
	−1	0	1
A Frying temperature (°C)	120	125	130
B Frying time (s)	900	1000	1100
C thickness (cm)	0.7	0.75	0.8

**Table 2:** Design and results for the central composition design.

Run	Frying Time	Temperature	Thickness	Hardness (N)	Δ*E*
1	−1	−1	−1	674.41 ± 78.84	24.81 ± 1.88
2	1	−1	−1	1494.56 ± 65.74	21.14 ± 2.74
3	−1	1	−1	747.70 ± 53.98	25.05 ± 3.84
4	1	1	−1	2525.37 ± 62.85	31.39 ± 2.05
5	−1	−1	1	1013.29 ± 48.05	29.57 ± 2.71
6	1	−1	1	2045.46 ± 70.30	26.46 ± 1.86
7	−1	1	1	358.78 ± 52.34	22.72 ± 2.33
8	1	1	1	2381.58 ± 62.08	29.42 ± 3.16
9	−1	0	0	1857.34 ± 73.10	21.76 ± 2.85
10	1	0	0	3227.36 ± 59.63	22.32 ± 3.11
11	0	−1	0	778.54 ± 55.24	27.97 ± 1.78
12	0	1	0	976.48 ± 57.49	30.12 ± 2.58
13	0	0	−1	1742.47 ± 56.10	25.40 ± 2.19
14	0	0	1	1825.02 ± 48.28	25.84 ± 3.23
15	0	0	0	1845.3 ± 38.96	24.91 ± 1.89

**Table 3:** Testing of the significance of the regression coefficients dependent variable.

Simulation Item	Hardness (N)			Δ*E*		
	Sum of Squares	*F* Value	*p* Value	Sum of Squares	*F* Value	*p* Value
Model	2.788 × 10^8^	1690.06	<0.0001 **	143.32	81.49	<0.0001 **
*A*	1.522 × 10^8^	8304.12	<0.0001 **	4.65	23.79	0.0046 **
*B*	2.986 × 10^6^	162.91	<0.0001 **	7.64	39.08	0.0015 **
*C*	5.965 × 10^5^	32.54	0.0023 **	3.86	19.76	0.0067 **
*AB*	1.464 × 10^7^	798.78	<0.0001 **	49.12	251.37	<0.0001 **
*AC*	8.062 × 10^5^	43.98	0.0012 **	0.1	0.52	0.5021
*BC*	7.807 × 10^6^	425.88	<0.0001 **	25.87	132.39	<0.0001 **
*A* ^2^	3.344 × 10^7^	1824.24	<0.0001 **	25.03	128.11	<0.0001 **
*B* ^2^	8.188 × 10^7^	4466.84	<0.0001 **	38.82	198.67	<0.0001 **
*C* ^2^	9.515 × 10^5^	51.91	0.0008 **	0.54	2.77	0.1571
Residual	91,654.65			0.985		
Lack of Fit	72,698.24	45.89	0.8631	0.46	36.98	0.385
Cor Total	2.789 × 10^8^			144.29		

Note: ** *p* < 0.01, the difference is extremely significant.

**Table 4:** Predicted and experimental values for the optimum technological.

Condition	Frying Temperature (°C)	Frying Time (s)	Thickness (cm)	Hardness (N)	Δ*E*
Model expected value	125.97	900.14	0.75	1988.00	21.14
Experimental actual value	130.00	900.00	0.75	2015.00 ± 48.17	23.27 ± 1.86

**Table 5:** Protein and fat contents of surimi cubes.

Type	Protein Content/g	Oil Content%
Vacuum deep frying (added with *Raphanus sativus*)	24.61 ± 0.34 ^c^	20.19 ± 0.06 ^b^
Atmospheric deep frying (added with *Raphanus sativus*)	22.42 ± 0.21 ^e^	11.31 ± 0.05 ^d^
Shallow frying (added with *Raphanus sativus*)	23.71 ± 0.28 ^d^	9.10 ± 0.06 ^f^
Vacuum deep frying (added without *Raphanus sativus*)	25.63 ± 0.22 ^b^	21.36 ± 0.10 ^a^
Atmospheric deep frying (added without *Raphanus sativus*)	24.32 ± 0.31 ^c^	13.48 ± 0.05 ^c^
Shallow frying (added with *Raphanus sativus*)	29.01 ± 0.29 ^a^	10.60 ± 0.05 ^e^

Different letters in the same chart represent significant differences between different treatments according to Duncan’s multiple range test. (*p* < 0.05).

## Data Availability

Not applicable.
